# Two Unusual Cases of Obstetrics

**Published:** 1889-08

**Authors:** H. McHatton

**Affiliations:** Macon, Ga.


### TWO UNUSUAL CASES OF OBSTETRICS.

By H. McHATTON, M. D., Macon, Ga.

Mrs. A., II Para. Normal labor at first confinement, having gone through her period of gestation with an unusual freedom from the minor troubles dependent on that condition, in excellent health, and spirits, was taken in labor at 5 P. M. Full dilation having taken place at 8, I ruptured the membranes; delivered at 9:15. Placenta by expression in 12 minutes; shortly after the delivery of the placenta, while my hand was on the firmly contracted uterus, she remarked in a very feeble voice that she was dying. To my astonishment I found that she was pulseless, and with cold extremities. Although I had the uterus in my hand, I could only convince myself that hemorrhage was not taking place by ocular demonstration. She rapidly went into a condition of extreme collapse, of that restless type, with sighing respiration usually associated with hemorrhage. When she could speak she constantly asked for water, and for several hours I thought she would die at any moment.

This condition was controlled by lowering the head, hypodermics of whiskey and morphia, aromatic spirits of ammonia, and hot applications. I did not dare leave the bedside till 7:30 A. M. Then she had a feeble circulation of 130; by evening her condition was fair, convalescence was good. Why did alarming and persistent shock occur in a case of this kind? The woman's health was good and the labor normal.

Mrs. X. came under my care about four years ago; she had had two induced abortions, and had been told that she could never bear a child. She was of rather a neurotic type, development good and general health fair.

Her urine was constantly loaded with pus, and had been so for years. The quantity was usually a little more than normal specific gravity 1015 to 1025, no sugar, and about the amount of albumen that you would expect with the pus. Repeated microscopical examinations covering a period of years, were negative, still the amount of pus was always sufficient to materially interfere with the examinations. The odor of the urine was constant and absolutely indescribable. Repeated physical examinations by myself and other physicians gave no clue to the cause of this condition; there was absolutely no clinical history in connection with it.

In due time she again became pregnant, and as I could see no reason for interfering, I let her go to term. Outside of considerable nausea for the first three months, her health was better than usual, but there was no change in the urinary condition. At full term she gave birth to a living child; labor and the puerperal period being normal. After confinement and until next conception her health was better than it had ever been. Gestation went on naturally until about the 8th month, when there was slight swelling of the feet and headache. No other important symptoms. Tr. Ferri Chloridi was ordered and improvement followed. On November 4th, about two weeks before her term, at 3 A. M., I was notified that she was suffering with her head; being detained with a case of obstetrics, I ordered ¼ gr. morph. sul. At 9 A. M. I called at her house, and was told that she had been sleeping quietly since 4 A. M. I found that the supposed sleep was coma. Her husband informed me that she had passed two small chambers full of urine of the usual type. Drs. Holt and Williams being called in consultation, we bled her. There was absolutely no effort at labor ; at 10 A. M. we began manual dilation and packed her head in ice; between 10 a m and 4 P. M. she had four convulsions ; took several hypodermics of whiskey, and had 6 oz. of urine drawn; delivered of a living child at 5 P. M., placenta following with next pain; pulse 125. Nov. 5th, 5 A. M., pulse 130; enema of whiskey, milk and egg. 8 A. M., 10 oz. of urine. 9 A. M., P. 135, T. 102; 10 gr. calomel, nutriment enema, with xxx gr. chloral.

2 P. M., P. 130, T. 103¼, urine 7 oz.; nutriment enema with xxx gr. bromide, and xx gr. chloral. 7 P. M., P. 130, T. 103, R. 35, x 92 calomel, enema, urine 8 oz. 10 P. M., P. 140, T. 104½ Tr. Digital, 15 m. hypodermically; copious involuntary discharge of urine. 11 P. M., pulse uncountable. Nov. 6th, 12 M., P. 140, T. 104, Digitalis. 4 A. M., enema. 7 A. M., involuntary discharge of urine. 8 A. M., P. 130, T. 104½, R. 35, enema. 9 A. M., P. 126, T. 104½, R. 28, Tr. digital, and 2 drops of croton oil. 11 A. M., P. 128, T. 105, R. 35, enema and xxx gr. quinine. 2 P. M., P. 138, T. 106, R. 42, digitalis. 4 P. M. enema with xxx gr. quinine. 5 P. M., P. 140, T. 107¼, R. 42; bowels and bladder moved involuntarily. Died at 6 P. M.

This case is certainly one of unusual interest. In the first place there was the constant and large amount of pus in the urine, covering a period of certainly four years; this condition being uninfluenced by pregnancy. There were no clinical phenomena, and repeated physical examinations by several of our best diagnosticians gave absolutely no clue to the pathological condition. The symptoms toward the last were in many respects those of uræmia; at the same time by referring to the history we find that at first at least the secretion was abnormally profuse. We hoped that by securing delivery we would get reaction, but although that was effected promptly and naturally, it had no beneficial effect, and from the time I first saw her, coma was absolute. A *post mortem* was not obtainable, and I have been unable to arrive at any satisfactory diagnosis.

